# Clinical image: multiple pulmonary, hepatic and Abdominal Wall hydatid cysts

**DOI:** 10.1093/omcr/omaf085

**Published:** 2025-06-27

**Authors:** Nahid Zaghba, Safia Rachid, Hanaa Harraz, Khadija Chaanoun, Hanane Benjelloun, Najiba Yassine

**Affiliations:** Pulmonology Department, IBN ROCHD University Hospital, 8, Lahcen El Arjoun district, Casablanca 20100, Morocco; Pulmonology Department, IBN ROCHD University Hospital, 8, Lahcen El Arjoun district, Casablanca 20100, Morocco; Pulmonology Department, IBN ROCHD University Hospital, 8, Lahcen El Arjoun district, Casablanca 20100, Morocco; Pulmonology Department, IBN ROCHD University Hospital, 8, Lahcen El Arjoun district, Casablanca 20100, Morocco; Pulmonology Department, IBN ROCHD University Hospital, 8, Lahcen El Arjoun district, Casablanca 20100, Morocco; Pulmonology Department, IBN ROCHD University Hospital, 8, Lahcen El Arjoun district, Casablanca 20100, Morocco

**Keywords:** hydatid cysts, pulmonary echinococcosis, disseminated cystic echinococcosis, liver cysts, Albendazole therapy, Thoracoscopic cystectomy

## Case report

A 27-year-old man presented with a 4-month history of purulent productive cough and progressive swelling of the left hypochondrium. The patient denied significant medical history but reported living in a rural area and regular exposures to livestock. Physical examination showed a palpable mass in the left hypochondrium.

**Figure 1:**
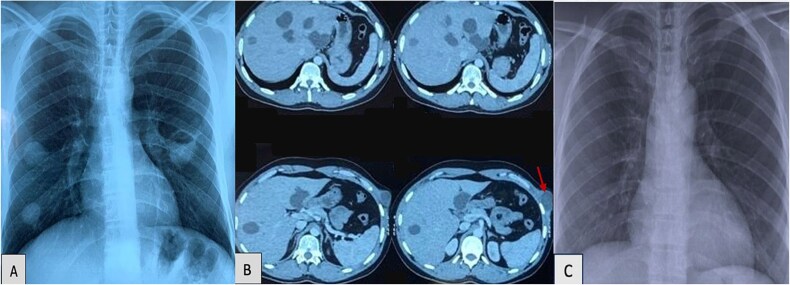
(**A**) Chest X-ray revealed multiple and bilateral hydatid cysts and a ruptured hydatid cyst in the left fowler. (**B**) Contrast-enhanced thoraco-abdominal-pelvic CT revealed multiple cystic lesions: At least eight cystic lesions in the liver, with the largest located at the junction of segments IV and VIII and a cystic lesion in the left external oblique muscle. (**C**) Normal follow-up image of chest X-ray.

The chest X-ray (Fig. 1A) revealed multiple well defined round opacities. Contrast-enhanced thoraco-abdominal-pelvic computed tomography CT (Fig. 1B) revealed multiple cystic lesions: Two cystic formations in the right lower lobe (Fowler region and anterobasal segment), measuring 25 mm and 25 × 23 mm, respectively. A lobulated cystic lesion was noted in the left Fowler region, measuring 37 × 36 mm. The cyst wall showed contrast enhancement and contained small air bubbles, suggesting a ruptured hydatid cyst and three smaller contiguous cystic lesions, measuring 15.5 × 6 mm, were found in the ventral segment of the culmen.

At least eight cystic lesions were seen in the liver, with the largest located at the junction of segments IV and VIII, measuring 40 × 42.3 mm and extending over 45 mm in height. A well-defined, hypodense cystic lesion was detected in the left external oblique muscle, measuring 30 × 40 mm and extending over 48 mm. The patient did not exhibit neurological symptoms and no neuroimaging (CT or MRI) was performed.

Laboratory investigations showed eosinophilia, and serologic testing for Echinococcus granulosus was strongly positive. Spirometry was within normal limits. Flexible bronchoscopy was performed and was unremarkable.

A diagnosis of disseminated hydatid disease was made based on these findings. The patient was initiated on oral albendazole at a dose of 400 mg twice daily for a total of 6 weeks before undergoing surgical intervention. Thoracoscopic resection of the pulmonary cysts was performed, followed by an open cystectomy for the hepatic and abdominal wall cysts. Albendazole was continued for 3 months. Follow-up image of Chest X-ray at 6 months was normal (Fig. 1C) and follow-up abdominal CT-scan is planned.

Hydatid disease is an endemic parasitic infection in regions such as North Africa, the Middle East, and parts of South America It is caused by Echinococcus granulosus that primarily affects the liver (55%–70%) followed by the lungs (18%–35%) [1]. Disseminated involvement are uncommon. Pulmonary cysts are typically asymptomatic unless they rupture or exert significant pressure on adjacent structures [2]. The presence of multiple cysts in the liver and lungs, along with an unusual abdominal wall cyst in this patient, represents a rare case of disseminated echinococcosis. The rare involvement of the external oblique muscle in this case, highlights the hematogenous or lymphatic spread potential of hydatid cysts to unusual sites [3].

Imaging and serologic tests are used to diagnose hydatid disease. CT is the gold standard imaging modality because it gives a detailed assessment about the number, size, and location of cysts [4].

Air-fluid level or internal septation may be seen in ruptured cysts. Normal spirometry and bronchoscopic findings, as in this case, can be useful in excluding obstructive airway disease or bronchial fistula [5].

Treatment includes antiparasitic therapy and surgical removal of cysts. Albendazole is the drug of choice and is given preoperatively to reduce the potential for cyst rupture and to prevent recurrence postoperatively. Surgical excision is the treatment of choice, and minimally invasive methods (thoracoscopy, laparoscopy) should be carried out whenever possible. A recent set of guidelines offers a tailored approach based on cyst size, location, and patient comorbidities. Uncomplicated cysts may respond to long-term therapy with albendazole, and most complex or ruptured cysts require surgical management [6].

Disseminated hydatid disease with multi-organ involvement including the abdominal wall is rare and presents a considerable diagnostic and therapeutic challenge. Best management requires a multidisciplinary approach with input from radiologists, pulmonologists, and surgeons. Prevention must be prioritized in particular in endemic regions.
